# Mechanism of Huo-Xue-Qu-Yu Formula in Treating Nonalcoholic Hepatic Steatosis by Regulating Lipid Metabolism and Oxidative Stress in Rats

**DOI:** 10.1155/2021/6026319

**Published:** 2021-04-29

**Authors:** Bin Cheng, Ai-Zhen Zhou, Wen Ge, Xiao-Min Yao, Juan Wang

**Affiliations:** ^1^School of Chinese Materia Medica, Zhejiang Pharmaceutical College, Ningbo 310053, China; ^2^Institute of Biopharmaceutical, Zhejiang Pharmaceutical College, Zhejiang, Ningbo 315100, China; ^3^School of Pharmacy, Zhejiang Pharmaceutical College, Ningbo 310053, China

## Abstract

Huo-Xue-Qu-Yu formula (HXQYF) is a prescription consisting of *Ginkgo biloba* leaf and *Paeonia lactiflora* Pall. for treating hyperlipidemia and NAFLD in China. Here, we investigated the hepatic and renal function, oxidative stress and lipid metabolism, and potential mechanisms of HXQYF on nonalcoholic fatty liver disease (NAFLD) rat models. NAFLD rat models were induced with high-fat diet (HFD) and 10% fructose water for 18 weeks and orally administered with or without HXQYF simultaneously. The results showed that HXQYF (22.5, 45, 90 mg/kg) significantly improved blood lipid levels *via* reducing serum TC, TG, LDL-C, and APOB values and elevating HDL-C and APOA1 levels in NAFLD rats. The higher levels of ALT, AST, CR, and BUN in serum induced by HFD were reduced by HXQYF. HE staining showed that HXQYF (90 mg/kg) reduced the accumulation of fat droplets and alleviated inflammatory response in liver cells. Three doses of HXQYF exhibited notable antioxidant effects by elevating SOD, GSH, and CAT activities and decreasing MDA and OH-1 levels in the liver. Furthermore, abnormal lipid metabolism caused by HFD was alleviated by HXQYF, which was associated with the upregulation of PPAR-*α*, AdipoR2, and CPT1 mRNAs as well as the downregulation of CYP2E1 and SREBP-1c mRNAs in liver tissue. In conclusion, our work verified that HXQYF could reduce the degree of hepatic steatosis, suppress oxidative stress, and attenuate lipid metabolism, thus preventing NAFLD.

## 1. Introduction

Nonalcoholic fatty liver disease (NAFLD), a most common chronic liver disease, arises from the modern lifestyle, ranging from simple steatosis to nonalcoholic steatohepatitis (NASH) and eventually to hepatocellular carcinoma (HCC) [1]. The increasing prevalence of NAFLD is associated with obesity, hypertension, and dyslipidemia, and diabetes [2, 3]. It increased the risk of developing diseases such as cirrhosis, extrahepatic cancers, cardiovascular disease, type 2 diabetes mellitus (T2DM), and chronic kidney disease [4].

Pathogenetic mechanisms of NAFLD are complexed and influenced by multiple factors, and a “multiple-hit pathogenesis” has been reported to explain it [5]. Based on the hypothesis, genetic factors, metabolic factors, and environmental factors were cooperated to promote the accumulation of fat in hepatocytes and successively cause inflammation and fibrosis [6]. Adipose tissue and muscle could promote inflammation and oxidative stress in the liver [7]. Excessive lipid accumulation in the liver plays an essential role in developing NAFLD; and the production of reactive oxygen species (ROS) is enhanced in both patients with and animal models of NAFLD [8, 9]. Therefore, it will be of interest to target on regulating lipid metabolism, oxidative stress, and inflammatory response for the prevention of the progression of NAFLD.

To date, various pharmacotherapy drugs for NAFLD have been revealed to have several side effects and poor long-term safety profiles in clinical trials [10]. Considerable studies on Chinese medicines have shown viable safe and beneficial treatment regimens to inhibit the progress of NAFLD [11]. It has been reported that about one-third of patients use herbal medicines or extracts in US liver clinics, and approximately 42% of Americans consume complementary and alternative medications annually [12].

In traditional Chinese medicine, the primary pathogenesis of NAFLD is the dysfunction of spleen in transportation and phlegm turbidity block. As the disease develops, the syndrome of blood stasis will be more prominent in patients. Blood stasis is not only the pathological product during the onset of NAFLD but also the motivating factor of its progression. Therefore, smoothing the liver and relieving depression, promoting blood circulation, and removing blood stasis are the main treatment principles of NAFLD [13]. A number of Chinese medicines with the action of activating blood and dissolving stasis, such as *Paeonia lactiflora*, *Ginkgo biloba* leaf, *Ligusticum chuanxiong*, and *Salvia miltiorrhiza*, have been proved with the pharmacological effects on regulating lipid metabolism, antioxidative stress, improving hemorheology, and antithrombosis [11, 14].

Several studies had been reported that *Ginkgo biloba* leaves showed widely pharmacological effects, such as antioxidant, anti-inflammatory, hepatoprotective effects, and cardiovascular protection [15–17]. It could regulate lipid metabolism and lessen the lipid accumulation in the livers of rats fed a high-fat diet at the transcriptome regulation level [18]. *Paeonia lactiflora* has exhibited multiple pharmacological effects, such as liver protection, antioxidation, vasodilatation of thoracic aorta, and antiallergic [19–21]. Studies confirmed that paeoniflorin protected against NAFLD in an animal model through anti-inflammatory effects [22], lowering lipid synthesis [23], promoting fatty acid oxidation, and improving insulin resistance [24].

Huo-Xue-Qu-Yu formula (HXQYF) is a prescription for treating hyperlipidemia and NAFLD, which is constituted by two Chinese herbal medicines including *Ginkgo biloba* leaf and *Paeonia lactiflora* Pall. [25, 26]. The *Ginkgo biloba* leaf has been used as traditional Chinese medicines for promoting blood circulation, removing stasis and turbidity, and lipid lowering [27]. The root of *Paeonia lactiflora* Pall. is applied for nourishing liver blood, promoting circulation, and removing stasis [27]. HXQYF not only can cool blood and invigorate circulation but also can remove stasis and reduce lipids [25].

Our previous work has demonstrated the lipid-lowering and hepatoprotective effects of HXQYF against rats with 18 weeks of high-fat diet (HFD) [25]. Currently, the dynamics of serum lipid levels and mechanism of HXQYF on NAFLD rat models remain uncertain. Therefore, this study aimed to evaluate the antisteatosis effects of HXQYF on HFD-induced NAFLD rats at the 4th, 6th, 10th, and 18th week. Furthermore, the underlying mechanism by which HXQYF protects against hepatic steatosis in NAFLD rats was explored.

## 2. Materials and Methods

### 2.1. Preparation and Phytochemical Analysis of HXQYF

The preparation and processing of HXQYF was the same as our previous study [26]. HXQYF was consistent of *Ginkgo biloba* leaf extract (GBE, 5 g) and *Paeonia lactiflora* extract (PLE, 4 g). GBE was purchased from Zhejiang Kangenbei Plant Extraction Co., Ltd. PLE was provided by Nanjing Zelang Pharmaceutical Technology Co., Ltd. GBE and PLE were mixed and ultrasonicated with distilled water (1L) for 2 h. Then, the mixture (HXQYF, 9.0 mg mL^−1^) was stored at 4°C.

The chemical profile analysis of HXQYF was conducted by HPLC. Briefly, 10 *μ*L of HXQYF and Ginkgo flavonoid standards (quercetin (**1)**, kaempferol (**2)**, isorhamnetin (**3**)) were injected into the HPLC system equipped with a DAD detector (Agilent 1290 Infinity; USA) and separated on the Agilent SB-C18 (4.6 × 250 mm, 5 *μ*m) column at 25°C. The mobile phase was methanol and 0.4% (v/v) phosphoric acid in H_2_O (50 : 50). The flow rate was 1 mL/min, and the monitoring wavelength was set at 360 nm.

HXQYF and *Ginkgo* terpenoid standards (ginkgolide A (**4**), B (**5**), and C (**6**) and bilobalide (**7**)) were injected into the HPLC system equipped with an ELSD detector (Agilent 1260; USA) and separated on the Agilent Zorbax XDB (4.6 × 250 mm, 5 *μ*m) column at 25°C. The mobile phase was methanol, tetrahydrofuran, and H_2_O (25 : 10 : 65). The flow rate was 1 mL/min. The drift tube temperature was 65°C, and the flow rate of carrier gas was 2.0 L/min.

HXQYF and paeoniflorin (**8**) standard were injected into the HPLC system equipped with a DAD detector (Agilent 1290 Infinity; USA) and separated on the Agilent SB-C18 (4.6 × 250 mm, 5 *μ*m) column at 25°C. The mobile phase was methanol and 0.05 mol/L monopotassium phosphate (40 : 65). The flow rate was 1 mL/min, and the monitoring wavelength was set at 230 nm.

### 2.2. Experimental Reagent and Apparatus

The kits for TC, TG, HDL-C, LDL-C, ALT, AST, APOA1, and APOB were supplied by Ningbo Meikang Medical System Biotechnology CO., Ltd. (Ningbo, China). The kits for SOD, GSH, CAT, MDA, and OH-1 were supplied by Nanjing Jiancheng Bioengineering Institute (Nanjing, China). An ACCUTE TBA-40FR fully automatic biochemistry analyzer was bought from Toshiba (Japan). A Nikon Eclipse Ti-SR inverted fluorescence microscope was bought from Nikon (Japan). The reverse transcription kit was bought from Bio SCI Co., Ltd. (Hangzhou, China).

### 2.3. Experimental Animals and Ethics Statement

The experimental procedures were conducted according to the Animal Care Ethics Committee of Zhejiang Pharmaceutical College (No. ZYLL2020004). Adequate measures were taken to minimize the pain of experimental animals. A total of 60 sexually mature male Sprague Dawley rats weighing 220–240 g were supplied by the Zhejiang Academy of Medical Sciences ([Certificate No. SCXK (Zhe) 2019-0002]). All rats were maintained under standard conditions: temperature 22 ± 2°C, relative humidity (50 ± 10%), and a 12-h light-dark cycle. Rats were acclimated for the first week by giving water *ad libitum* and chow pellets in the laboratory environment.

### 2.4. Animal Experimental Design

The NAFLD rat model was established by HFD and 10% fructose water for 18 weeks. HFD containing 76.5% standard diet, 12% lard, 1% cholesterol, 5% yolk powder, 5% whole milk powder, and 0.5% bile sodium was prepared in our laboratory.

Totally 60 male SD rats were randomly divided into 6 groups: 1) normal control group, 2) HFD group, 3–5) HXQYF (22.5, 45, and 90 mg/kg) groups, and 6) positive control group ESS (136.8 mg/kg). The normal control group was fed with a normal diet and distilled water, and the other groups were fed with HFD and 10% fructose water (2 mL/100 g, i.g., once daily). Rats in the positive control group were given ESS (136.8 mg/kg, i.g., once daily). Rats in HXQYF groups were given HXQYF (22.5, 45, and 90 mg/kg, i.g., once daily). All administrations were conducted for 18 weeks. Diet and water were provided *ad libitum*. The body weight of each rat was measured every week.

### 2.5. Serum Biochemical Analysis

At the 4th, 6th, 10th, and 18th week, after overnight fasting following the last drug administration, 0.5 mL blood was taken from the orbital vein under mild pentobarbital anesthesia of each rat. Blood samples were centrifuged at 3,500 rpm for 10 min to obtain the serum. Serum lipid profiles (TC, TG, HDL-C, LDL-C) and liver enzymes (AST, ALT) analyses were performed using a fully automatic biochemistry analyzer. Serum levels of APOA1, APOB, SCR, BUN, and UA were measured with a fully automatic biochemistry analyzer at the 18th week. Atherosclerosis index (AI) = serum (TC − HDL)/HDL.

### 2.6. Liver Histopathology

Hepatic tissue samples were fixed in 10% neutral buffered formalin and embedded in paraffin. 3 mm-thick sections were cut using rotary microtome (Leica Biosystems, Wetzlar, Germany) and then stained with hematoxylin-eosin (HE) staining to observe the size and number of fat globules in the liver. The slides were photographed *via* ×20 and ×100 objective lenses to assess the histopathological changes [28].

### 2.7. Determination of Antioxidant Enzymes

Hepatic tissue samples (100 mg) were homogenized with 0.9% (w/v) NaCl solution to obtain the supernatants. The activities of SOD, MDA, GSH, CAT, and OH-1 in whole-tissue supernatants were determined using commercially available kits according to the manufacturer's instructions. Values were expressed as milligrams of protein.

### 2.8. Real-Time PCR

Frozen liver tissues (100 mg) were powdered and then mixed with 1 mL Trizol reagents (Invitrogen) for total RNA extraction, according to the manufacturer's protocol. cDNA was synthesized from equal amounts of total RNA using the reverse transcription kit (Bio SCI, China). Real-time quantitative PCR was performed in 20 *μ*L reactions to detect the expressions of SREBP-1c, PPAR-*α*, AdipoR2, CPT1, and CYP2E1 mRNAs in hepatic tissues using Agilent Strata gene Mx3005P. Amplification was performed with the following protocol: 40 cycles (10 s at 95°C, 10 s at 58°C, and 10 s at 72°C) after an initial activation step for 1 min at 94°C. The housekeeping gene *β*-actin was used as an internal control. Primer sequences are shown as follows: SREBP-1c (101 bp) forward primer: GCTGGACCACAGAAAGGTG, reverse primer: TGGCAGTTGATGTAGAGGCTA; PPAR-*α* (120 bp) forward primer: GCTTCATCACCCGAGAGTTC, reverse primer: GGGAAATGTCACTGTCATCCA; AdipoR2 (127 bp) forward primer: ATCTGTGTGCTGGGCATTG, reverse primer: GCAAGGTAGGGATGATTCCA; CPT1 (92 bp) forward primer: ACAGTGGGACATTCCAGGAG, reverse primer: AGGAATGCAGGTCCACATCA; CYP2E1 (97 bp) forward primer: TCTGCTCCTGTCTGCTATTCTG, reverse primer: ACTGCCAAAGCCAACTGTGA; *β*-actin (105 bp) forward primer: GCTCTCTTCCAGCCTTCCTT, reverse primer: GGTCTTTACGGATGTCAACG.

### 2.9. Statistical Analysis

All values were analyzed using SPSS, version 22.0. Data were expressed as mean ± standard deviation (SD). Comparisons among the 6 groups were performed using the one-way analysis of variance (ANOVA) compared with Dunnett's multiple or post hoc analysis. Significant differences were considered at *P* < 0.05, and higher significance was at *P* < 0.01.

## 3. Results

### 3.1. Identification of Bioactive Components of HXQYF

The HPLC chromatograms of reference standards and HXQYF samples are shown in Supplementary Material Figure S1-1to S1-3. Eight chromatographic peaks were identified in the phytochemical profile of HXQYF, including quercetin (**1)**; kaempferol (**2)**; isorhamnetin (**3**); ginkgolide A (**4**), B (**5**), and C (**6**); bilobalide (**7**); and paeoniflorin (**8**). All standards were well separated and showed good selectivity within 30 min. The identification of the components in HXQYF was based on comparisons of their retention times (*t*_R_), UV spectra, and chromatograms with those of each standard. The Ginkgo flavonoid and terpenoid contents in HXQYF in the present study were 3.75% and 1.3%, respectively. Paeoniflorin in HXQYF was 3.61%.

### 3.2. Effects of HXQYF on Rats' Body Weight (BW)

**Figure 1:**
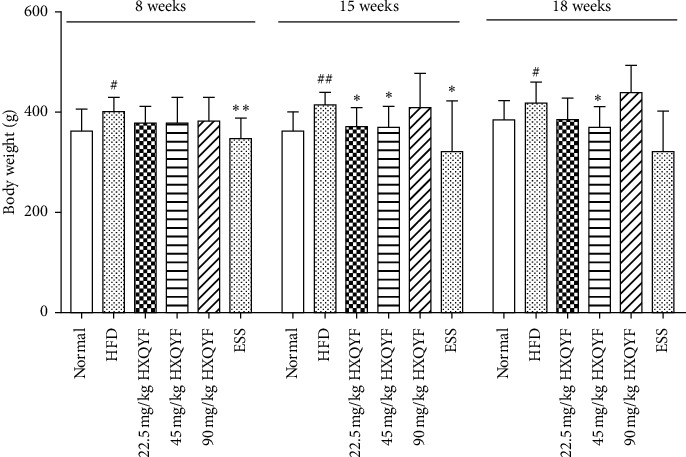
Effects of HXQYF on body weight (BW) during the experiment period. Rats were fed with a high-fat diet (HFD) to establish a mouse model of nonalcoholic fatty liver (NAFLD). The NAFLD rats were administered with HXQYF (22.5, 45, 90 mg/kg). Essentiale (ESS) was adopted as a positive control for comparison. The BW of rats was measured in different weeks until the end of the experiment. BW values given are in means ± SD, with *n* = 10. ^#^*P* < 0.05 and ^##^*P* < 0.01 versus normal. ^*∗*^*P* < 0.05 and ^*∗∗*^*P* < 0.01 versus HFD. Statistically significant differences were determined using one-way ANOVA followed by Dunnett's multiple comparisons test or post hoc analysis.

To determine the effect of HXQYF on obesity-related factor, the BWs of rats were measured once a week until the end of the experiment. As shown in Figure 1, significant differences of BWs were observed in the HFD-fed group, compared with the normal control after feeding with HFD for 8 weeks (*P* < 0.05). After administration of HXQYF for 15 weeks, the BW of NAFLD rats was significantly reduced, compared with the HFD group (*P* < 0.05).

### 3.3. Effects of HXQYF on Serum Lipid Levels and AI in Serum at 4–18 Weeks

**Figure 2:**
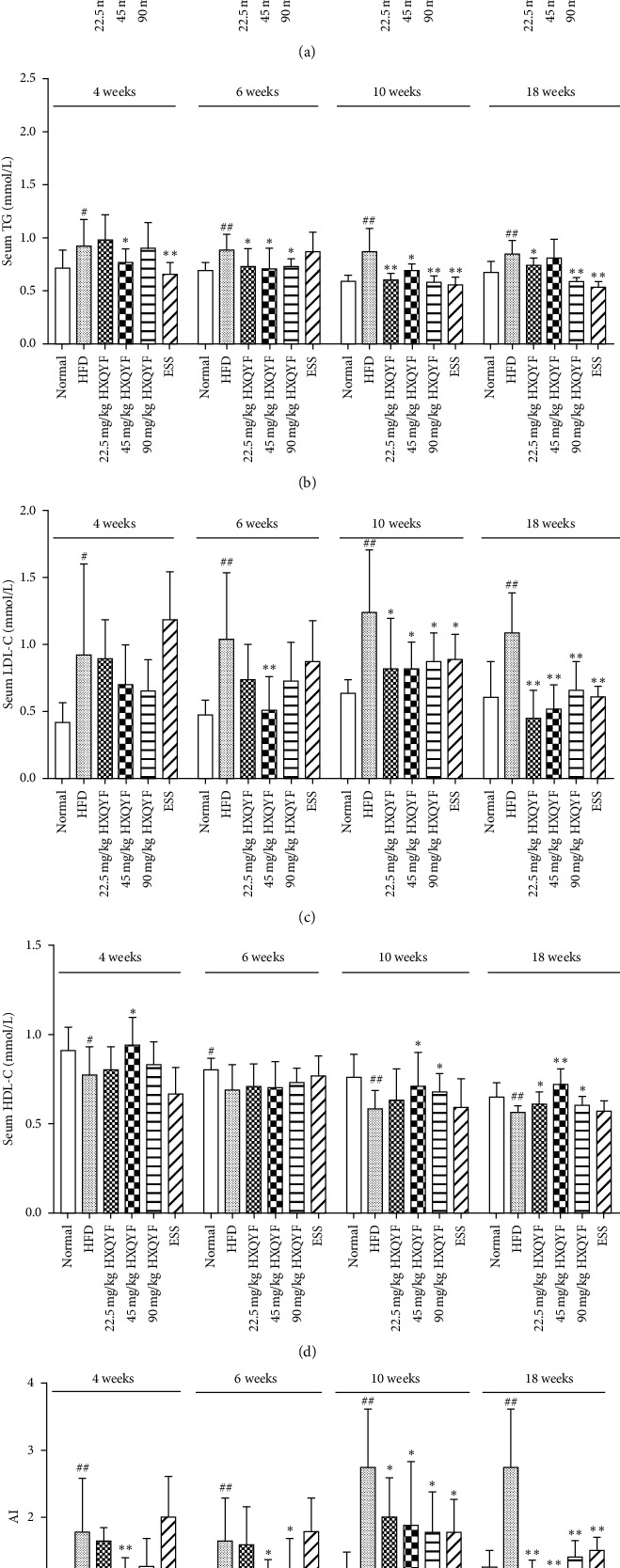
Effects of HXQYF on (a) TC, (b) TG, (c) LDL, and (d) HDL levels and (e) AI value in serum at 4–18 weeks. Experimental details were described in Figure 1. After 18 weeks of treatment, serum total cholesterol (TC), triglyceride (TG), high-density lipoprotein (HDL), and low-density lipoprotein (LDL) levels were measured. Values given are in means ± SD, with *n* = 10. ^#^*P* < 0.05 and ^##^*P* < 0.01 versus normal. ^*∗*^*P* < 0.05 and ^*∗∗*^*P* < 0.01 versus HFD. Statistically significant differences were determined using one-way ANOVA followed by Dunnett's multiple comparisons test or post hoc analysis.

The contents of TC, TG, LDL-C, and HDL-C in serum play a notable role in the analysis of lipid metabolism. To explore the effect of HXQYF in lipid metabolism of NAFLD rats, serum lipid levels were investigated, and the results are shown in Figure 2(a)–2(d). The serum levels of TG, TC, LDL-L, and AI in the HFD-fed group were significantly higher than those in the normal control group at 4–18 weeks (*P* < 0.05 or *P* < 0.01). In contrast, the serum level of HDL-L was significantly lower than that in the normal control group at 4–6 (*P* < 0.05) and 10–18^th^ (*P* < 0.01) weeks.

Compared with the HFD-fed group, NAFLD rats treated with HXQYF (45 and 90 mg/kg) showed a significant decrease in serum TC and LDL-C levels at the 6th, 10th, and 18th week (*P* < 0.05 or *P* < 0.01), respectively. The TG level and AI value (Figure 2(e)) were significantly reduced by HXQYF (22.5, 45, 90 mg/kg) from 4 to 18 weeks (*P* < 0.05), respectively. On the contrary, NAFLD rats treated with HXQYF (45 and 90 mg/kg) showed a significant increase in HDL-C at the 4th and 10th week (*P* < 0.05) and markedly elevated at the 18th week (*P* < 0.01). The positive ESS drug (136.8 mg/kg) treatment significantly reduced serum TC, TG, LDL-C, and AI at the 10th (*P* < 0.05) and 18th (*P* < 0.01) week and increased the HDL-C level only at the 10th week (*P* < 0.05).

### 3.4. Effects of HXQYF on Liver and Renal Functions

**Figure 3:**
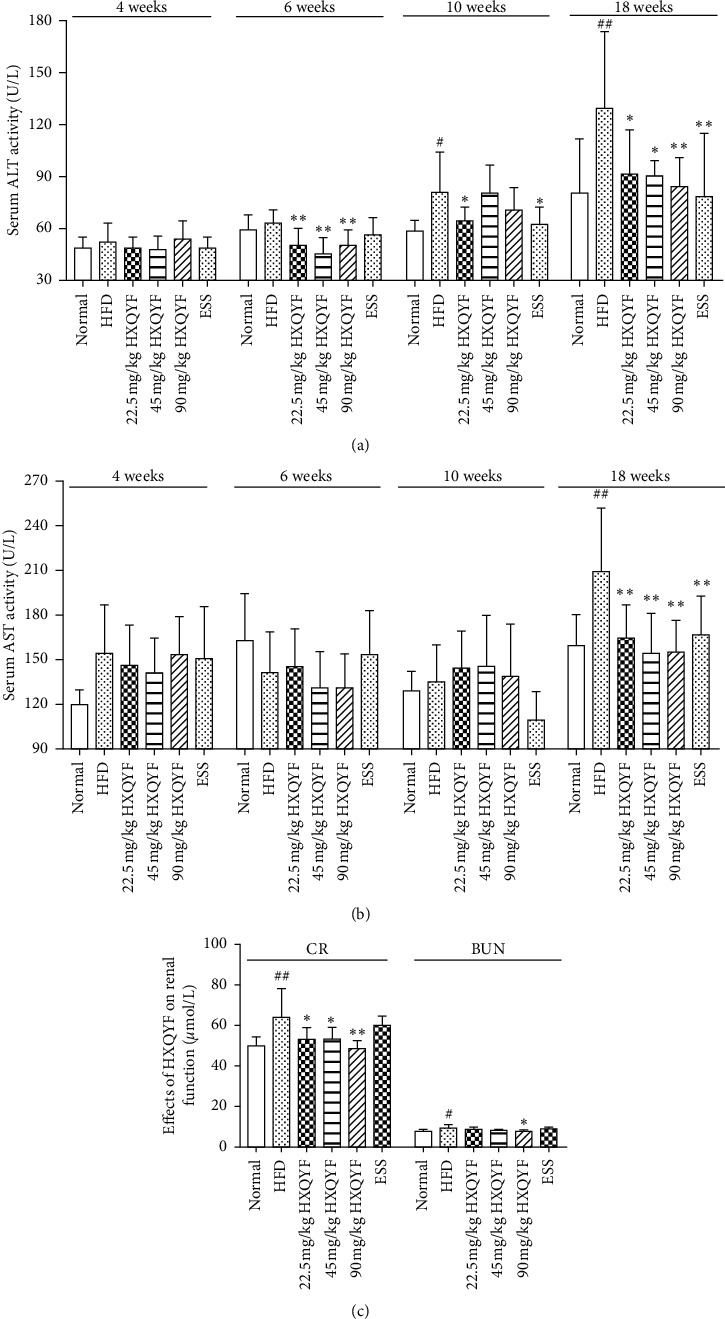
Effects of HXQYF on liver and renal function. Experimental details are described in Figure 1. After treatment with HXQYF (22.5, 45, 90 mg/kg), serum ALT and AST at 4–18 weeks and serum CR and BUN at the 18th week were measured. Values given are in means ± SD, with *n* = 10. ^#^*P* < 0.05 and ^##^*P* < 0.01 versus normal. ^*∗*^*P* < 0.05 and ^*∗∗*^*P* < 0.01 versus HFD. Statistically significant differences were determined using one-way ANOVA followed by Dunnett's multiple comparisons test or post hoc analysis.

To determine the hepatoprotective and renal protection effects of HXQYF, NAFLD rats were treated with different doses of HXQYF. As shown in Figure 3, compared with the normal control group, NAFLD rats showed elevated serum levels of ALT (Figure 3(a)), AST (Figure 3(b)), CR (Figure 3(c)), and BUN (Figure 3(c)) (*P* < 0.05), respectively, which suggested that HFD caused hepatic and renal injury in NAFLD rats. The elevated levels of serum ALT, AST, CR, and BUN were significantly reduced in the groups administered with HXQYF, especially at 90 mg/kg, at the 18th week. ESS (136.8 mg/kg) treatment significantly reduced serum ALT and AST levels from the 10th week (*P* < 0.05), but it showed no significant effect on serum CR and BUN (*P* > 0.05).

### 3.5. Histopathological Observations of Rat Livers

**Figure 4:**
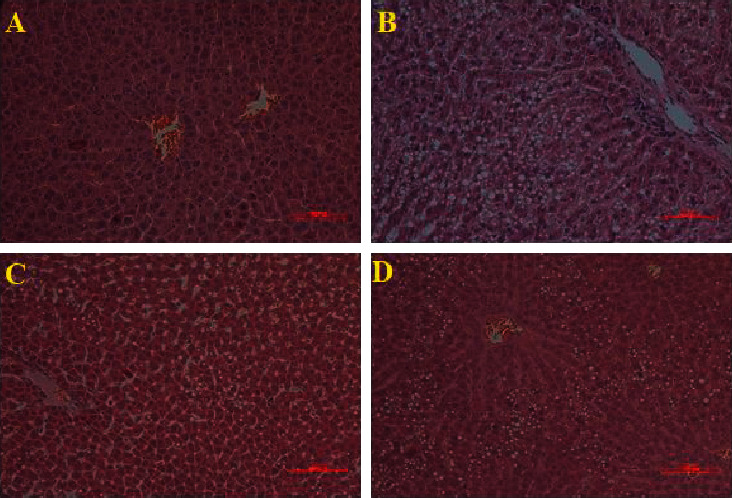
Effects of HXQYF on histopathological changes of the hepatic tissue at the 18th week. Experimental details are described in Figure 1. After treatment with HXQYF for 18 weeks, histopathological changes of the hepatic tissue analyzed by image Pro-Plus 7200 software. HE staining, 200x magnification. (a) Normal control group. (b) HFD-fed group. (c) HXQYF (90 mg/kg) group. (d) ESS (136.8 mg/kg) group.

Histopathological lesions were graded according to Brunt's criteria [29] by an experienced histopathologist. As shown in Table 1, scores of steatosis and lobular inflammation were higher in NAFLD rats compared with the normal control group. The administration of HXQYF reduced the score in NAFLD rats when compared with the HFD group. Furthermore, liver sections of normal rats showed intact structure, and distinct hepatic cells were arranged radially in the central vein with the prominent nucleus and distinct nucleolus (Figure 4(a)). Sections of hepatic tissues in the HFD-fed group showed hepatic vacuoles, large fat droplets in the cytoplasm of hepatocytes, and inflammatory cell infiltrations in the lobule and portal areas (Figure 4(b)). In the HXQYF treatment groups, especially the HXQYF (90 mg/kg) group, the fat droplets were reduced, lipid degeneration and inflammatory response were significantly alleviated, and the liver cell volume became smaller compared with the HFD-fed group (Figure 4(c)).

### 3.6. Effects of HXQYF against Oxidative Stress in NAFLD Rats

To explore the role of HXQYF against oxidative stress, we examined the hepatic levels of SOD, GSH, CAT, MDA, and OH-1. As shown in Table 2, the activities of SOD (*P* < 0.01), GSH (*P* < 0.01), and CAT (*P* < 0.05) in the liver of NAFLD rats were significantly decreased, whereas MDA and OH-1 levels were markedly increased compared with those of the normal control group (*P* < 0.01). Compared with the HFD-fed group, oral administration of HXQYF (22.5, 45, 90 mg/kg) significantly raised the activities of SOD, GSH, and CAT but inhibited MDA and OH-1 formation in the liver of NAFLD rats (*P* < 0.01 or *P* < 0.05).

### 3.7. Effects of HXQYF on the Balance of Cholesterol Transport

**Figure 5:**
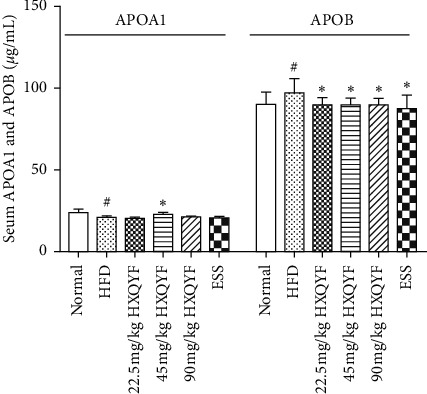
Effects of HXQYF on APOA1 and APOB levels in serum at the 18th week. Experimental details are described in Figure 1. After 18 weeks of treatment with HXQYF (22.5, 45, 90 mg/kg), APOA1 and APOB were measured. Values given are in means ± SD, with *n* = 10. ^#^*P* < 0.05 and ^##^*P* < 0.01 versus normal. ^*∗*^*P* < 0.05 and ^*∗∗*^*P* < 0.01 versus HFD. Statistically significant differences were determined using one-way ANOVA followed by Dunnett's multiple comparisons test or post hoc analysis.

The levels of APOA1 and APOB reflect the balance of cholesterol transport [30]. The results in Figure 5 showed that APOA1 (*P* < 0.05) was significantly decreased in serum of HFD-fed rats, while APOB was on the contrary. However, the serum APOA1 level was increased by HXQYF (45 mg/kg) administration (*P* < 0.05); the serum APOB level was significantly decreased when treated with three doses of HXQYF (*P* < 0.05). ESS (136.8 mg/kg) only decreased the value of APOB in serum at the 18th week.

### 3.8. Effects of HXQYF on Gene Expression in the Liver of NAFLD Rats

**Figure 6:**
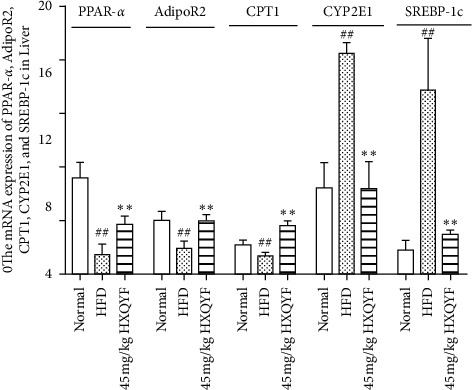
The mRNA expressions of PPAR-*α*, AdipoR2, CPT1, CYP2E1, and SREBP-1c in normal, HFD, and HXQYF (45 mg/kg) groups. Relative abundance of mRNA expression was calculated by the well-recognized 2^−△Ct^ method. Each bar or dot and error bar represents mean ± SD (*n* = 6). ^#^*P* < 0.05 and ^##^*P* < 0.01 versus normal. ^*∗*^*P* < 0.05 and ^*∗∗*^*P* < 0.01 versus HFD. Statistical significant differences were determined using one-way ANOVA followed by Dunnett's multiple comparisons test or post hoc analysis.

As shown in Figure 6, the mRNA expressions of PPAR-*α*, AdipoR2, and CPT1 in the liver of NAFLD rats were significantly downregulated (*P* < 0.05), while the expressions of SREBP-1c and CYP2E1 were markedly upregulated (*P* < 0.01), as compared with the normal control group. HXQYF (45 mg/kg) upregulated PPAR-*α*, AdipoR2, and CPT1 mRNA expressions compared with the HFD group, and the increase of AdipoR2 and CPT1 mRNA expressions were similar to the normal control (*P* < 0.01). The upregulated expressions of SREBP-1c and CYP2E1 were decreased by HXQYF (45 mg/kg), and the decrease of CYP2E1 was similar to the normal control (*P* < 0.01).

All of the above data are summarized in Tables S3-1 and S3-2.

## 4. Discussion

NAFLD is one of the most common causes of chronic liver injury, affecting both adults and children [31, 32]. It ranges from simple fatty liver or nonalcoholic steatohepatitis (NASH) to fibrosis, cirrhosis, and liver failure [33]. The earliest stage of NAFLD is simple accumulation of excessive triglycerides in hepatocytes [34]. Studies have shown that high consumption of HFD causes health problems, such as hypertriglyceridemia, hepatic steatosis, and liver damage [35]. Therefore, feeding a high-fat, cholesterol-rich diet has been proposed in the experiment to induce hyperlipidemia and NAFLD. In the present study, the NAFLD rat model was established by HFD and 10% fructose water. NAFLD rats showed elevated serum levels of TC, TG, and LDL-C and a reduced HDL-C value from the 8th week to the end of the experiment. Furthermore, HE staining showed severe fatty degeneration in the liver of the HFD-fed group, which indicated that the model was successfully established.

HXQYF consisted of GBE and PLE. Based on the pharmacological properties of *Ginkgo biloba* leaf and *Paeonia lactiflora* Pall. described above, the effects of HXQYF on treating NAFLD were assessed in the present study. The increased serum levels of TC, TG, and LDL-C and a decrease in HDL-C are major risk factors for NAFLD [36]. To analyze the possible role of HXQYF on lipid metabolism, serum lipid levels of HFD rats were investigated. After oral administration with HXQYF for 4 weeks, the serum TG level was significantly reduced and the HDL-C level was raised; whereas TC and LDL-C levels were decreased from the 6^th^ week. At the end of the experiment, HXQYF remarkably ameliorated on those serum lipids. APOA1 is the main protein component of HDL, which promotes the transportation of cholesterol and regulates the metabolism of HDL [37]. One research demonstrated the protective role of ApoA1 in a diet-induced fatty liver animal model [38]. ApoB is the main protein component of VLDL and LDL, which is responsible for transporting cholesterol in LDL to tissues and plays a central role in lipid metabolism [39]. In this study, NAFLD rats treated with HXQYF for 18 weeks showed a higher level of serum APOA1 and lower ApoB, which were coincidence with the HDL-C and LDL-C levels in rats treated with HXQYF. Therefore, HXQYF could promote the transportation of cholesterol and regulate HDL-C and LDL-C levels to prevent and treat NAFLD. These findings suggested that HXQYF treatment might be useful to prevent NAFLD *via* improving lipid metabolism from the 4th week.

Disorders of lipid metabolism caused lipid accumulation in hepatocytes [40]. Meanwhile, reactive oxygen species (ROS) induce inflammatory reactions, resulting in hepatocyte damage [41]. In this research, NAFLD rats showed elevated serum levels of ALT and AST compared with those in the normal control group, suggesting that HFD caused hepatic injury in NAFLD rats. However, HXQYF could attenuate those increases, which was coincided with the histological changes shown in Figure 4. Histopathological findings showed that HXQYF ameliorated the disturbed liver architecture and decreased fat droplets in various parts of the liver. These results indicated that HXQYF was effective in improving HFD-induced liver function, preventing hepatic steatosis and alleviating hepatocyte damage induced by oxidative stress and inflammation.

Numerous epidemiological evidence implicated that NAFLD is an independent risk factor for chronic kidney disease (CKD), and the associated factors such as metabolic syndrome, dysbiosis, and unhealthy diet could also contribute to the mechanisms linking NAFLD and CKD [42]. The anomalies could be associated to an escalated risk of CKD in adults strongly recommending the reduced intake of fatty diets to prevent renal lipotoxicity. In animal studies, mice fed with HFD for sixteen weeks were persuaded to lipid deposition in kidney and caused renal damage [43]. Similarly, studies on adult rats showed that extravagant intake of dietary fats impaired normal kidney functioning [44]. In the present research, rats fed with HFD for 18 weeks showed elevated CR and BUN levels in serum, which exhibited the abnormal renal function in rats. However, higher levels of serum CR and BUN were decreased by HXQYF (90 mg/kg), which suggested the benefiting renal protection of HXQYF on NAFLD rats.

Our previous studies reported that HXQYF could benefit against hypercholesterolemia and NAFLD risk factors by regulating blood lipids and anti-inflammatory properties [25]. The current study further demonstrated the contributions of HXQYF to lipid metabolism and antioxidant effects in liver tissue. In the liver, the increase in free fatty acids (FFA) and lipid overload are critical components that could increase ROS and decrease the antioxidant system in NAFLD [45]. FFA oxidation is known as a crucial source of ROS in fatty livers. ROS could attack polyunsaturated fatty acids and trigger the lipid peroxidation in cells, leading to the formation of aldehyde by-products, including MDA [46]. Oxidative stress plays a central role in the pathogenesis of NAFLD and is considered the most crucial mechanism causing damage to the liver [47]. Here, we explored the role of HXQYF in the regulation of oxidative status in rats with NAFLD through examining the liver SOD, CAT, GSH, MDA, and OH-1 activities. The results suggested that liver contents of SOD, GSH, and CAT were remarkably decreased in HFD-fed rats, whereas values of MDA and OH-1 were elevated. Notably, the whole process showed above were reversed by oral administration of HXQYF for 18 weeks, especially at 90 mg/kg, which indicated the antioxidant activity of HXQYF to ameliorate NAFLD.

CYP2E1, an index of oxidant and toxic substance levels, could oxidize various small toxic substrates including xenobiotics, fatty acids, etc [48]. Previous studies have shown that in CYP2E1 knockout mice, the adipose tissue glucose uptake and insulin suppression of hepatic glucose output were enhanced [49]. In the present study, the mRNA expression of CYP2E1 in NAFLD rat models was increased, which was similar to the result reported by Abdelmegeed, et al. [48]. HXQYF (45 mg/kg) resulted in the expression of CYP2E1 in the liver as low as the normal group. SREBP-1c is a key transcription factor in regulating hepatic lipogenesis [50]. Some research studies illustrated that fructose intake strongly accelerated lipogenesis by upregulating the expression of SREBP-1c [51]. In line with previous studies, the mRNA expression of SREBP-1c was significantly increased in NAFLD rats; however, it was downregulated by HXQYF (45 mg/kg), accompanied by the decrease of lipid accumulation in the liver. This result was similar to other reports in NAFLD rats induced by high-fat diet [22].

PPAR-*α* plays a vital role in regulating lipid metabolism and inflammation [52]. It is involved in hepatic lipogenesis, ketogenesis, fatty acid transport, and oxidative stress in hepatic tissues [53]. AdipoR2 was proved to be involved in the PPAR-*α* pathway, and the upregulation of which could inhibit hepatic inflammatory response and oxidative stress in mice [54, 55]. As a rate-limiting enzyme in the *β*-oxidation of fatty acids, CPT1 is the downstream target of the PPAR-*α* pathway, which is involved in lipid metabolism [56]. In this study, the results showed that expressions of AdipoR2, PPAR-*α*, and CPT1 mRNAs in the liver of NAFLD rats were significantly downregulated, which were reversed by treatment with HXQYF (45 mg/kg) in NAFLD rats.

HXQYF mainly consists of flavonoid components (quercetin, kaempferol, isorhamnetin), terpenoid (Ginkgolide A), and paeoniflorin, which have been confirmed potential for treating NAFLD *via* reducing the hepatic lipid accumulation, causing mitochondrial oxidative stress, inhibiting *de novo* lipid synthesis, and regulating the ROCK/IRS/Akt signaling pathways [57–59]. The effects of HXQYF on NAFLD rats in this study were similar to the above description.

Taken together, HXQYF upregulated the mRNA expressions of PPAR-*α*, AdipoR2, and CPT1 and down-regulated the mRNA expressions of CYP2E1 and SREBP-1c in the liver to promote fat oxidation and lipid decomposition and inhibit cholesterol synthesis and lipid accumulation, thereby regulating lipid metabolism disorders. The data of this study indicated that HXQYF inhibited oxidative stress, ameliorated liver and renal function, as well as alleviated lipid metabolism through different signaling pathways, attenuating NAFLD.

## 5. Conclusion

In conclusion, Ginkgo flavonoid and terpenoid contents including quercetin, kaempferol, isorhamnetin, ginkgolide A/B/C and bilobalide, and paeoniflorin were found in HXQYF. The anti-NAFLD effects of HXQYF might be achieved by the synergistic action of these compounds. HXQYF controlled body weight gain, alleviated oxidative stress, improved liver and renal dysfunction, reduced hepatic fat accumulation, and alleviated lipid metabolism disorders associated with NAFLD. Therefore, HXQYF has great potential to alleviate the progression of NAFLD in future.

## Figures and Tables

**Table 1:** Effects of HXQYF on morphological changes in NAFLD rats' liver tissues.

Groups	Dose (mg/kg)	Steatosis					Inflammatory cells infiltration			
		0	1	2	3	4	0	1	2	3
Normal	—	8	—	—	—	—	6	2	—	—
HFD	—	—	—	1	5	2	—	4	2	2
HXQYF	90	1	4	2	1	—	1	6	1	—
ESS	136.8	—	2	5	1	—	1	5	2	—

Criteria of histopathological scores are as follows: fat: hepatocytes fat containing less 5% (score 0); hepatocytes fat containing 5% to 30% (score 1); hepatocytes fat containing 31% to 50% (score 2); hepatocytes fat containing 51% to 75% (score 3); and hepatocytes fat containing more than 75% (score 4). Lobular inflammation: no inflammation and necrosis (score 0); mild injury/inflammation (score 1); moderate injury/inflammation (score 2); and marked injury/inflammation (score 3). *n* = 8 in each group.

**Table 2:** Effects of HXQYF against oxidative stress in NAFLD rats.

Groups	Dose (mg·kg^−1^)	SOD (U/mL)	GSH (*μ*mol/g prot)	CAT (U/mg prot)	MDA (nmol/mg prot)	HO-1 (U/L^)^
Normal	—	88.00 ± 7.92	25.11 ± 4.92	192.04 ± 61.53	17.66 ± 6.73	38.77 ± 2.99
HFD	—	72.03 ± 8.90^##^	17.72 ± 6.58^##^	151.32 ± 24.22^#^	26.06 ± 5.26^##^	43.19 ± 1.54^##^
HXQYF	22.5	79.85 ± 8.87^∗^	19.06 ± 2.69	176.39 ± 32.23^*∗*^	13.73 ± 4.05^*∗∗*^	42.73 ± 1.24
	45	83.18 ± 7.35^*∗∗*^	22.23 ± 3.95^*∗*^	216.60 ± 44.79^*∗∗*^	12.79 ± 2.43^*∗∗*^	44.24 ± 3.53
	90	80.99 ± 11.13^*∗*^	24.53 ± 4.63^*∗∗*^	228.27 ± 43.80^*∗∗*^	16.30 ± 6.32^*∗∗*^	46.09 ± 3.23^*∗*^
ESS	136.8	71.28 ± 10.62	20.52 ± 2.82	240.84 ± 32.11^*∗∗*^	15.18 ± 4.30^*∗∗*^	42.79 ± 2.66

The NAFLD rats were administered with the HXQYF (22.5, 45, 90 mg/kg). ESS was adopted as a positive control for comparison. After 18 weeks of treatment, superoxide dismutase (SOD), glutathione (GSH), catalase (CAT), monochrome display adapter (MDA), and Heme oxygenase-1 (HO-1) were detected. Values given are in means ± SD, with *n* = 10. ^#^*P* < 0.05 and ^##^*P* < 0.01 versus normal. ^*∗*^*P* < 0.05 and ^*∗∗*^*P* < 0.01 versus HFD. Statistically significant differences were determined using one-way ANOVA followed by Dunnett's multiple comparisons test or post hoc analysis.

## Data Availability

The data used to support the fndings of this study are available from the first author, and included within the article and supplementary information files.

## Abbreviations

AdipoR2: Adiponectin receptor 2
ALT: Alanine aminotransferase
AI: Atherosclerosis index
AST: Aspartate aminotransferase
APOA1: Apolipoprotein A1
APOB: Apolipoprotein B
CAT: Catalase
CPT1: Carnitine palmitoyltransferase 1
CYP2E1: Cytochrome P4502E1
GSH: Glutathione
HO-1: Heme oxygenase-1
HFD: High-fat diet
HXQYF: Huo-Xue-Qu-Yu formula
HDL-C: High-density lipoprotein cholesterol
HE: Hematoxylin-eosin
LDL-C: Low-density lipoprotein cholesterol
MDA: Monochrome display adapter
NAFLD: Nonalcoholic fatty liver disease
PPAR-*α*: Peroxisome proliferator-activated receptor-*α*
SOD: Superoxide dismutase
SREBP-1c: Sterol regulatory element-binding protein
TC: Total cholesterol
TG: Triglyceride.

## Supplementary Materials

Supplementary Materials Supplemental Figure S1: HPLC chromatograms of HXQYF aqueous extract. Supplemental Table S3-1 and S3-2: a summary on the effects of HXQYF on serum lipids in NAFLD rats at different weeks.
