# Supplementary material for: Mechanism of Huo-Xue-Qu-Yu Formula in Treating Nonalcoholic Hepatic Steatosis by Regulating Lipid Metabolism and Oxidative Stress in Rats
Source: Evid Based Complement Alternat Med. 2021 Apr 29;2021:6026319. doi: 10.1155/2021/6026319 (PMC8102110; doi:10.1155/2021/6026319)
Supplement: Supplementary Materials — Supplemental Figure S1: HPLC chromatograms of HXQYF aqueous extract. Supplemental Table S3-1 and S3-2: a summary on the effects of HXQYF on serum lipids in NAFLD rats at different weeks. [file 6026319.f1.docx]

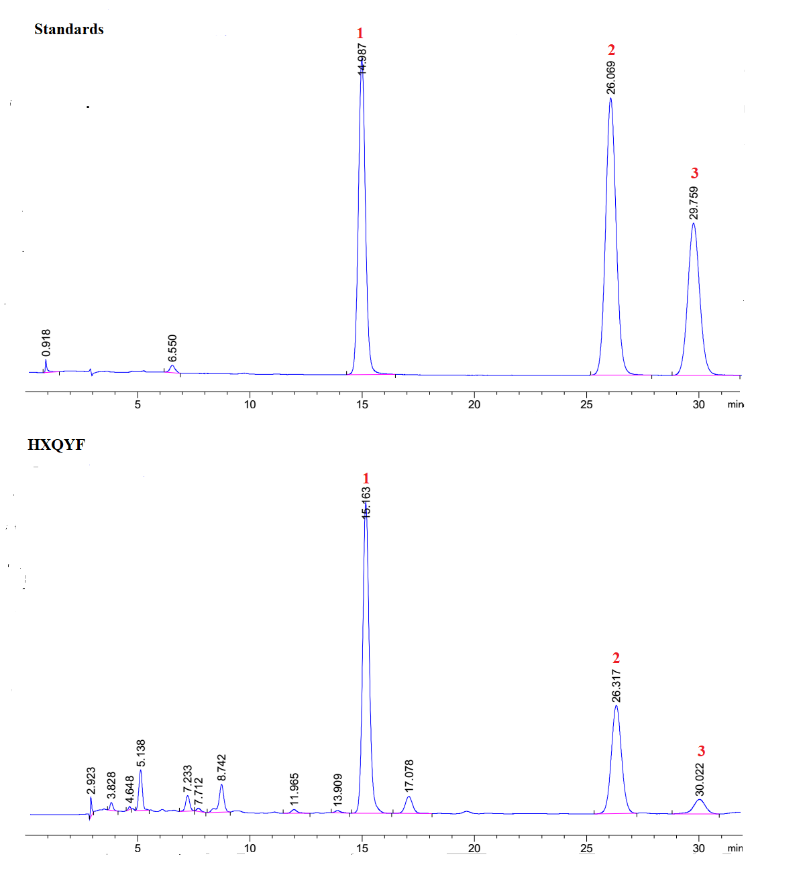


**Figure S1-1 HPLC chromatograms of Ginkgo flavonoid standards and HXQYF.** Ginkgo flavonoid standards: quercetin (1), kaempferol (2), isorhamnetin (3).


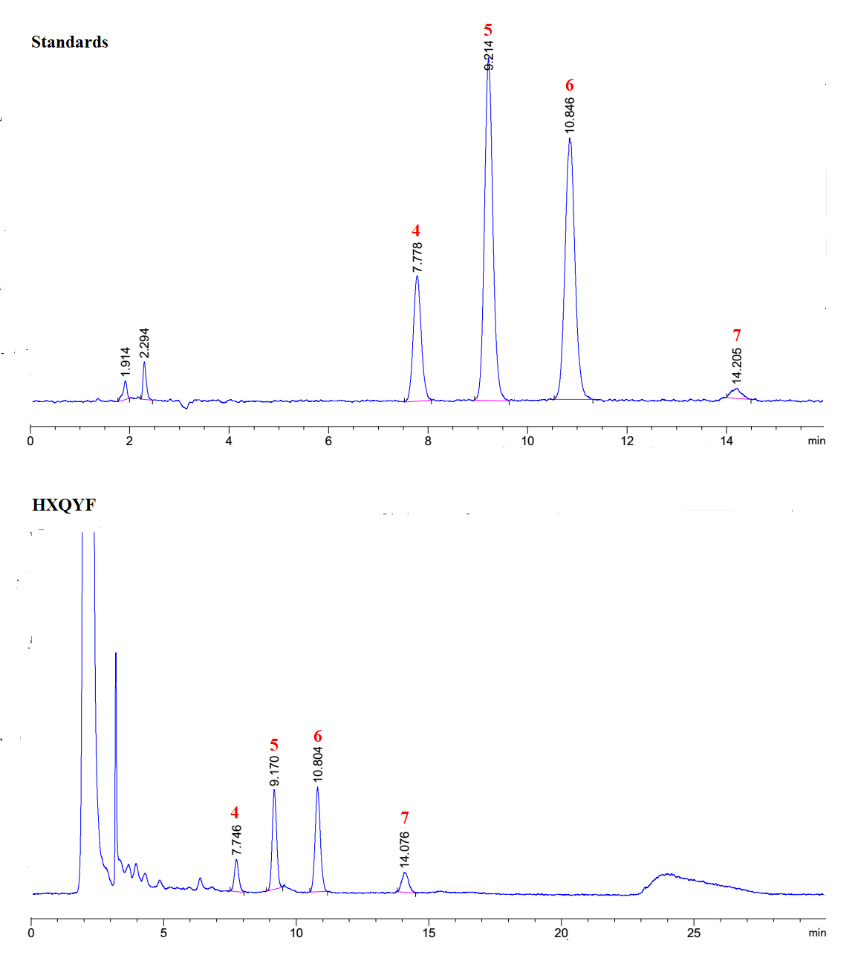


**Figure S1-2 HPLC chromatograms of Ginkgo terpenoid standards and HXQYF.** Ginkgo terpenoid standards: ginkgolide A(4), B(5), C(6), bilobalide (7).


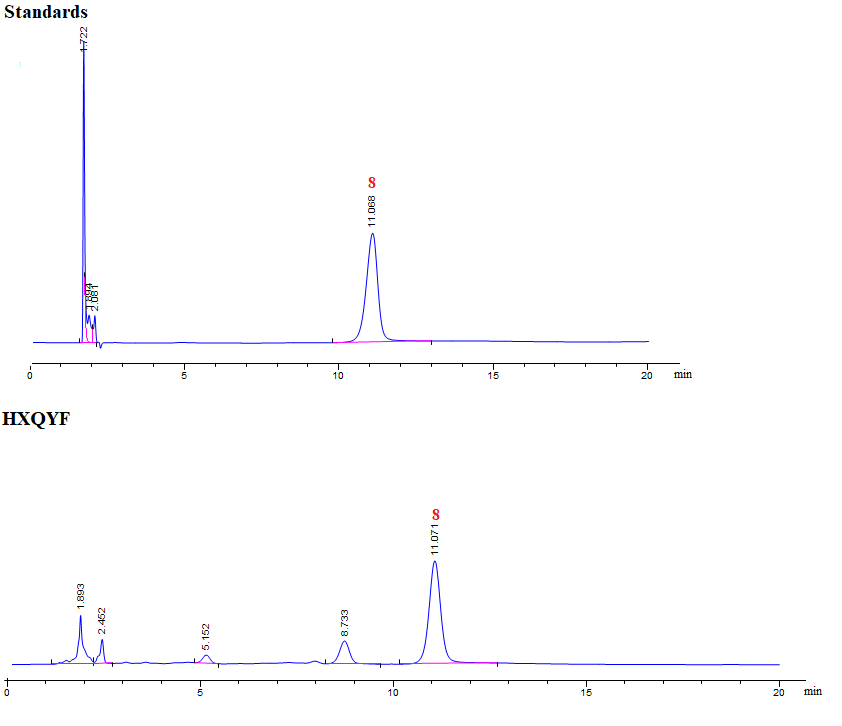


**Figure S1-3 HPLC chromatograms of paeoniflorin standard (8) and HXQYF.**

**Table S3-1 A summary on the effects of HXQYF on serum lipids in NAFLD rats**

|  | 4^th^ week | | | 6^th^ week | | | 10^th^ week | | | 18^th^ week | | |
| --- | --- | --- | --- | --- | --- | --- | --- | --- | --- | --- | --- | --- |
| HXQYF (mg/kg) | 22.5 | 45 | 90 | 22.5 | 45 | 90 | 22.5 | 45 | 90 | 22.5 | 45 | 90 |
| Serum TC | — | — | — | — | ↓* | — | — | — | ↓* | ↓** | ↓** | ↓** |
| TG | — | ↓* | — | ↓* | ↓* | ↓* | ↓** | ↓* | ↓** | ↓* | — | ↓** |
| HDL | — | ↑* | — | — | — | — | — | ↑* | ↑* | ↑* | ↑** | ↑* |
| LDL | — | — | — | — | ↓** |  | ↓* | ↓* | ↓* | ↓** | ↓** | ↓** |
| AI | — | ↓** | — | — | ↓* | ↓* | ↓* | ↓* | ↓* | ↓** | ↓** | ↓** |
| ALT | — | — | — | ↓** | ↓* | ↓* | ↓* | — | — | ↓* | ↓* | ↓** |
| AST | — | — | — | — | — | — | — | — | — | ↓** | ↓** | ↓** |

↑: increased or elevated; ↓: decreased or inhibited; —: unaltered; /: untested. ^∗^*p* < 0.05, ^∗∗^*p* < 0.01 versus HFD.

**Table S3-2 A summary on the effects of HXQYF on experimental NAFLD rats**

|  | 18 weeks | | |
| --- | --- | --- | --- |
| HXQYF (mg/kg) | 22.5 | 45 | 90 |
| Serum SCR | ↓* | ↓* | ↓** |
| BUN | — | — | ↓* |
| APOA1 | — | ↑* | — |
| APOB | ↓* | ↓* | ↓* |
| Liver SOD | ↑* | ↑** | ↑* |
| GSH | — | ↑* | ↑** |
| CAT | ↑* | ↑** | ↑** |
| MDA | ↓** | ↓** | ↓** |
| OH-1 | — | — | ↑* |
| PPAR-*α* | — | ↑** | — |
| AdipoR2 | — | ↑** | — |
| CPT1 | — | ↑** | — |
| CYP2E1 | — | ↓** | — |
| SREBP-1c | — | ↓** | — |
| BW | — | — | ↑* |

↑: increased or elevated; ↓: decreased or inhibited; —: unaltered; /: untested. ^∗^*p* < 0.05, ^∗∗^*p* < 0.01 versus HFD.
